# The relationship between cancer and medication exposure in patients with systemic lupus erythematosus: a nested case-control study

**DOI:** 10.1186/s13075-020-02228-6

**Published:** 2020-06-26

**Authors:** Jinyan Guo, Zhigang Ren, Jianhao Li, Tianfang Li, Shengyun Liu, Zujiang Yu

**Affiliations:** 1 Department of Rheumatology and Immunology, The First Affiliated Hospital of Zhengzhou University, No. 1, Jianshe East Road, Zhengzhou, 450052 Henan Province China; 2 Department of Infectious Disease, The First Affiliated Hospital of Zhengzhou University, No. 1, Jianshe East Road, Zhengzhou, 450052 Henan Province China

**Keywords:** Lupus, Cancer, Autoantibody, Disease activity, Hydroxychloroquine

## Abstract

**Background:**

Systemic lupus erythematosus (SLE) is associated with increased risk of cancer and the mechanism remains unclear. Here, we examined the level of auto-antibodies and disease activity index scores in SLE patients with cancers and analyzed whether medications for SLE management might contribute to the higher cancer risk in SLE patients.

**Methods:**

In this retrospective study, we carried out a nested case-control study in a large cohort of SLE patients. We screened 5858 SLE patients to identify the newly diagnosed and yet to be treated cancers. The following clinical features were evaluated: auto-antibodies levels, SLE disease activity index scores, and previous medication used for SLE management. Systemic glucocorticoid, cyclophosphamide, hydroxychloroquine (HCQ), methotrexate, and azathioprine were considered the main medication indices.

**Results:**

Our analyses identified 51 SLE patients who also had cancer and 204 matched control patients who had SLE but not cancer. Of the 51 SLE patients, thyroid cancer (14/51, 27.45%), cervical cancer (10/51, 19.61%), and lung cancer (7/51, 13.73%) were the most common types. Our analyses did not reveal any significant differences in the levels of auto-antibodies in SLE patients with cancers relative to the control group. Further, we observed that disease activity was significantly lower in SLE patients with cancers relative to the matched control SLE group. There was no statistically significant association between the cancer risk and the use of systemic glucocorticoid, cyclophosphamide, methotrexate, or azathioprine. Importantly, the administration of HCQ was significantly lower in SLE patients suffering cancers relative to the cancer-free matched control group.

**Conclusions:**

Our analyses indicate that SLE patients with cancers might have a lower disease activity at the time of cancer diagnosis. HCQ was negatively associated with cancer risk in SLE patients. These findings highlight a potential and novel prevention strategy for SLE.

## Background

Systemic lupus erythematosus (SLE) is an autoimmune inflammatory disorder characterized by an aberrant production of auto-antibodies and a wide range of clinical manifestations and complications. Antinuclear antibodies (ANAs) refer to a broad class of antibodies targeting a wide range of cellular and nuclear components. These class of antibodies are generated as a result of loss of immune tolerance. Anti-double-stranded DNA antibody (anti-dsDNA) and anti-Sm antibody (anti-Sm) are the important hallmarks of SLE [1]. For patients presenting with SLE, treatment with hydroxychloroquine (HCQ) is recommended unless contraindicated. In cases where the disease affects major organs or present refractory symptoms, treatment with systemic glucocorticoid (GC), cyclophosphamide (CTX), methotrexate (MTX), or azathioprine (AZA) is recommended. As early diagnosis and advanced treatments have significantly improved the survival, malignancies are becoming an important cause of mortality in SLE patients [2–10]. However, the mechanism underlying such an increase in cancer risk is not completely understood.

Although they are important serological markers of autoimmune disease, ANAs are not unique to autoimmune disorders and multiple studies have reported the involvement of ANAs in a variety of neoplastic diseases [11, 12]. Interestingly, several lines of evidence suggested that ANAs have anti-neoplastic effects in cancer patients without concomitant autoimmune diseases [13, 14] and are associated with a better prognosis [15–17]. A previous report showed that the damage index, defined by the Systemic Lupus International Collaborating Clinics/American College of Rheumatology (SLICC/ACR), is associated with overall cancer risk [18]. Contradictory findings were observed in another study in which no association was found between the adjusted mean SLE Disease Activity Index 2000 (SLEDAI-2 K) and the risk of lymphoma [19]. Similarly, the relationship between the use of immunosuppressant and cancer risk in SLE is inconsistent. A previous report showed that the application of CTX and AZA did not increase cancer risk in SLE patients [20]. Another study demonstrated that although immunosuppressants including CTX, AZA, and MTX were not associated with overall cancer risk, they may increase the risk of hematological malignancies in patients with SLE [18]. It has been reported that CTX administration is associated with increased cancer risk while administration of HCQ is thought to lower cancer risk [21].

To improve our understanding of the relationship between cancer risk and SLE pharmacologic interventions, we carried out a nested case-control study. To this end, we analyzed clinical features including auto-antibodies and disease activity in 5858 SLE patients as well as the pharmacologic interventions used in the management of SLE. Our results showed that SLE patients with cancers had lower disease activity and that HCQ was negatively associated with cancer risk in these patients.

## Methods

### Study design

All patients recruited into this retrospective cohort study met the updated American College of Rheumatology criteria for the classification of SLE [22]. All patients included in the study were hospitalized at the First Affiliated Hospital of Zhengzhou University between October 1, 2010, and October 1, 2019. Any SLE patients younger than 18 years old or less than 18 years at SLE diagnosis age were excluded from the study. Cancer diagnosis was confirmed by histological analyses, and any patients diagnosed with premalignant lesions were excluded. Patients with a concomitant diagnosis of rheumatoid arthritis, SjÖgren syndrome, inflammatory myopathy, autoimmune hepatitis, or primary biliary cholangitis were defined as having overlap syndrome. Participants were assigned into a cancer group and control group. This study was approved by the Ethical Committee of the First Affiliated Hospital of Zhengzhou University, ethical approval number no.2019-KY-199 (Additional file 1).

### Clinical and laboratory examinations

The following patient information was collected: age, gender, age at SLE diagnosis, and course of SLE progression as well as chronic comorbidities including hypertension, diabetes mellitus, and dyslipidemia. In addition, the positive rate and titers of autoantibodies were assessed including antinuclear antibody (ANA) and other autoantibodies such as anti-dsDNA, anti-Sm, anti-Ro52 antibody (anti-Ro52), anti-Ro60 antibody (anti-Ro60), anti-SSB antibody (anti-SSB), anti-nucleosome antibody (anti-Nuc), anti-histone antibody (anti-His), anti-ribosome antibody (anti-Rib), and anti-nRNP antibody (anti-nRNP). The SLE Disease Activity Index (SLEDAI) scores were calculated at the day of cancer diagnosis as previously described [23]. The information on the use of GC, HCQ, CTX, MTX, and AZA was also collected from the date of SLE diagnosis to the date of cancer diagnosis for participants in the cancer group or the date of admission for those in the control group.

### Propensity score matching

Propensity score matching was performed to minimize selection bias when evaluating the effect of immunosuppressant on cancer risk. Based on the propensity scores, each participant with cancer was matched with four cancer-free participants. The propensity score was calculated by taking into account the following variables: age, gender, age at SLE diagnosis, disease course of SLE and comorbidities such as hypertension, diabetes mellitus, and dyslipidemia.

### Statistical analysis

Patient clinical features and SLEDAI scores were compared between the cancer group and the control group using an independent-sample *t* test for continuous variables or the chi-square test for categorical variables. Conditional logistic regression analysis was used for the evaluation of the association between cancer odds and medical intervention with pharmacologic agents. Cancer occurrence was treated as a dependent variable in the logistic analysis. Associations were firstly evaluated without consideration for confounding factors followed by an analysis taking into account such factors (Table 1). SPSS statistical software version 20.0 was used to conduct data analysis and propensity score matching [SPSS Inc., Chicago, IL].

**Table 1:** Characteristics of patients in the cancer and control groups

Characteristics	Before matching			After matching		
	Cancer group (*n* = 51)	Control group (*n* = 5497)	*P* value	Cancer group (*n* = 51)	Control group (*n* = 204)	*P* value
Age, median	47	35	< 0.0001	47	46	0.69
Female gender, *n* (%)	49, 96.08	4930, 89.68	0.2055	49, 96.08	192,94.12	0.74
Age at SLE diagnosis, median	41	33	0.0024	41	39	0.96
Disease course of SLE, median	60	6	< 0.0001	60	60	0.92
Hypertension	5, 9.80%	199, 3.620%	0.0195	5, 9.80%	15, 7.35%	0.56
Diabetes mellitus	5, 9.80%	189, 3.438%	0.0375	5, 9.80%	14, 6.86%	0.55
Dyslipidemia	8, 15.38%	494, 8.18%	0.0969	8, 15.38%	28, 13.72%	0.72

## Results

### Patient characteristics

**Fig. 1:**
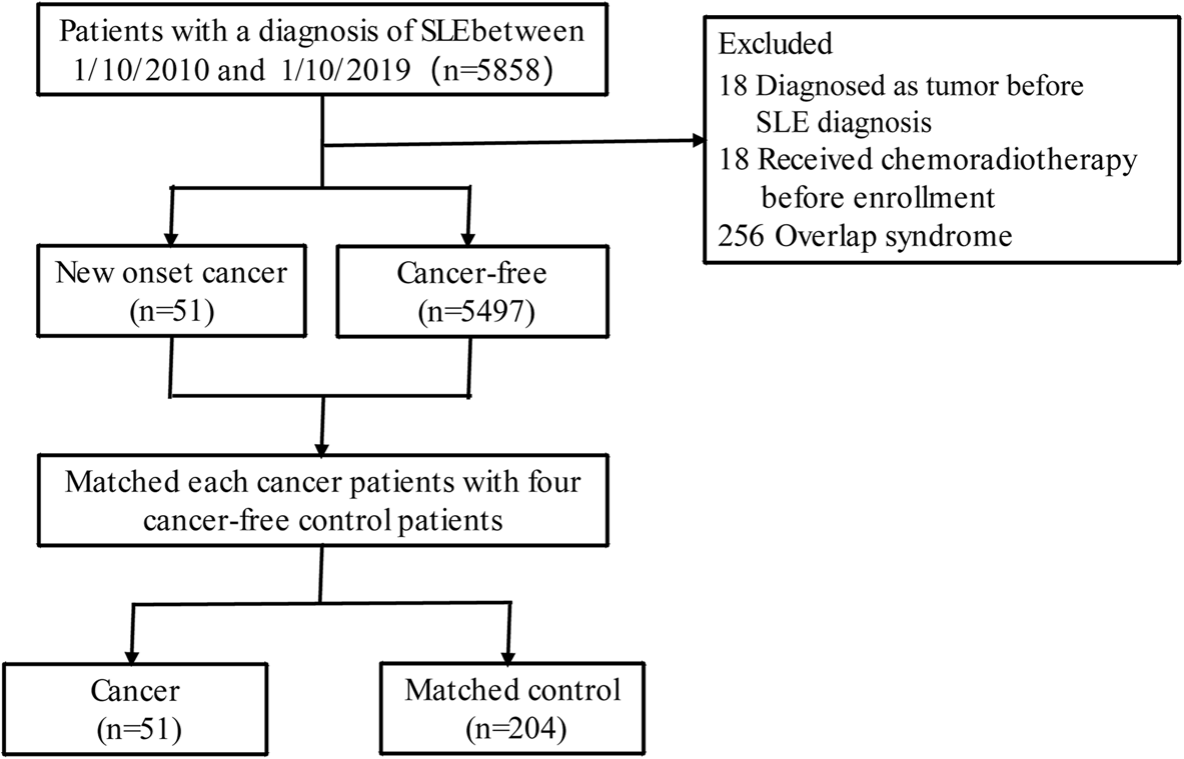
Flow chart of the study design

A total of 5858 patients diagnosed with SLE between October 1, 2010, and October 1, 2019, were recruited into this study. Eighteen patients that had been diagnosed with cancer prior to SLE diagnosis, 18 patients that had metastasis or received chemotherapy prior to hospital admission, and 274 patients with overlap syndrome were excluded from subsequent analyses. Of the 5548 patients that were eligible for further analyses, 51 were cancer patients while the remaining 5497 were cancer-free patients. Each cancer case was matched with four cancer-free patients. Our study therefore consisted of 51 cancer patients and 204 matched cancer-free patients (Fig. 1).

Patients’ characteristics of the cancer group and the control group are showed in Table 1. Before matching, patients in the cancer group were older, diagnosed with SLE at a more advanced age, and had a longer disease course of SLE and a higher prevalence of comorbidities. However, such difference was not clear after matching (Table 1).

### Distribution of all cancers and specific cancer types

The specific types of cancer are showed in Table 2. Four patients had hematological cancer (2 leukemia and 2 non-Hodgkin’s lymphoma). No patient had Hodgkin’s lymphoma in this cohort. A total of 47 SLE patients had non-hematological cancer, with thyroid cancer being the most frequently observed type of cancer (27.45%), followed by cervical cancer (19.61%) and lung cancer (13.73%).

**Table 2:** Specific types of cancers in the cancer cohort

Sites and types	*N* (%)
Hematological cancer	
Leukemia	2 (3.92%)
Non-Hodgkin’s lymphoma	2 (3.92%)
Non-hematological cancer	
Reproductive system	
Cervical cancer	10 (19.61%)
Vulvar cancer	2 (3.92%)
Uterus cancer	1 (1.96%)
Non-reproductive system	
Thyroid cancer	14 (27.45%)
Lung cancer	7 (13.73%)
Gastric carcinoma	3 (5.89%)
Rectal carcinoma	2 (3.92%)
Hepatic carcinoma	1 (1.96%)
Appendix cancer	1 (1.96%)
Bile duct cancer	1 (1.96%)
Pancreatic cancer	1 (1.96%)
Renal cell cancer	2 (3.92%)
Breast cancer	2 (3.92%)

### Level of auto-antibodies in the cancer and control groups

The levels of auto-antibodies in both groups are summarized in Table 3. No significant differences were observed between the two groups in the positive rate of ANA, anti-dsDNA, anti-Sm, anti-RO52, anti-RO60, anti-SSB, anti-Nuc, anti-His, anti-Rib, and anti-nRNP.

**Table 3:** Comparison of levels of auto-antibodies between the two groups

Auto-antibodies	Cancer group (*n* = 51) (positive/total, percentage)	Control group (*n* = 204) (positive/total, percentage)	*P* value
ANA	37/37, 100%	198/200, 99.00%	1.00
Anti-dsDNA	15/35, 42.86%	95/180, 52.78%	0.36
Anti-Sm	5/36, 13.89%	33/172, 19.19%	0.63
Anti-RO52	26/37, 70.27%	110/172, 63.95%	0.57
Anti-RO60	20/37, 54.05%	110/172, 63.95%	0.57
Anti-SSB	4/36, 11.11%	16/172, 9.30%	0.76
Anti-Nuc	11/36, 30.56%	70/172, 40.70%	0.35
Anti-His	7/36, 19.44%	50/172, 29.07%	0.31
Anti-Rib	10/36, 27.78%	58/172, 33.72%	0.56
Anti-nRNP	11/36, 30.56%	64/172, 37.21%	0.57

*ANA* antinuclear antibody, *anti-dsDNA* anti-double-stranded DNA antibody, *anti-Sm* anti-Sm antibody, *anti-RO52* anti-RO52 antibody, *anti-RO60* anti-RO60 antibody, *anti-SSB* anti-SSB antibody, *anti-Nuc* anti-nucleosome antibody, *anti-His* anti-histone antibody, *anti-Rib* anti-ribosome antibody, *anti-nRNP* anti-nRNP antibody

### SLEDAI and disease activity indexes in cancer and control groups

The SLEDAI and disease activity indexes in cancer and control groups are showed in Table 4. Patients in the control group always had a higher percentage of decreased C3 and elevated proteinuria than in the cancer group (82.22% vs 44.44%, *P* < 0.01; 36.00% vs 12.24%, *P* < 0.01; respectively). The SLEDAI was higher in the control group than that in the cancer group (8 vs 2, *P* < 0.01). No significant differences in low C4, low white blood cell, and thrombocytopenia were observed between the two groups.

**Table 4:** Comparison of SLEDAI between the two groups

Indicator	Cancer group (*n* = 51) (positive/total, percentage)	Control group (*n* = 204) (positive/total, percentage)	*P* value
Low C3	16/36, 44.44%	74/90, 82.22%	< 0.01
Low C4	14/36, 38.89%	48/90, 53.33%	0.17
Low WBC	6/48, 12.50%	26/102, 25.49%	0.09
Low PLT	10/48, 20.83%	34/102, 33.33%	0.13
Proteinuria	6/49, 12.24%	36/100, 36.00%	< 0.01
SLEDAI, median	2	8	< 0.01

*WBC* white blood cell, *PLT* platelet, *SLEDAI* SLE Disease Activity Index

### Medication exposure and cancer risk

**Fig. 2:**
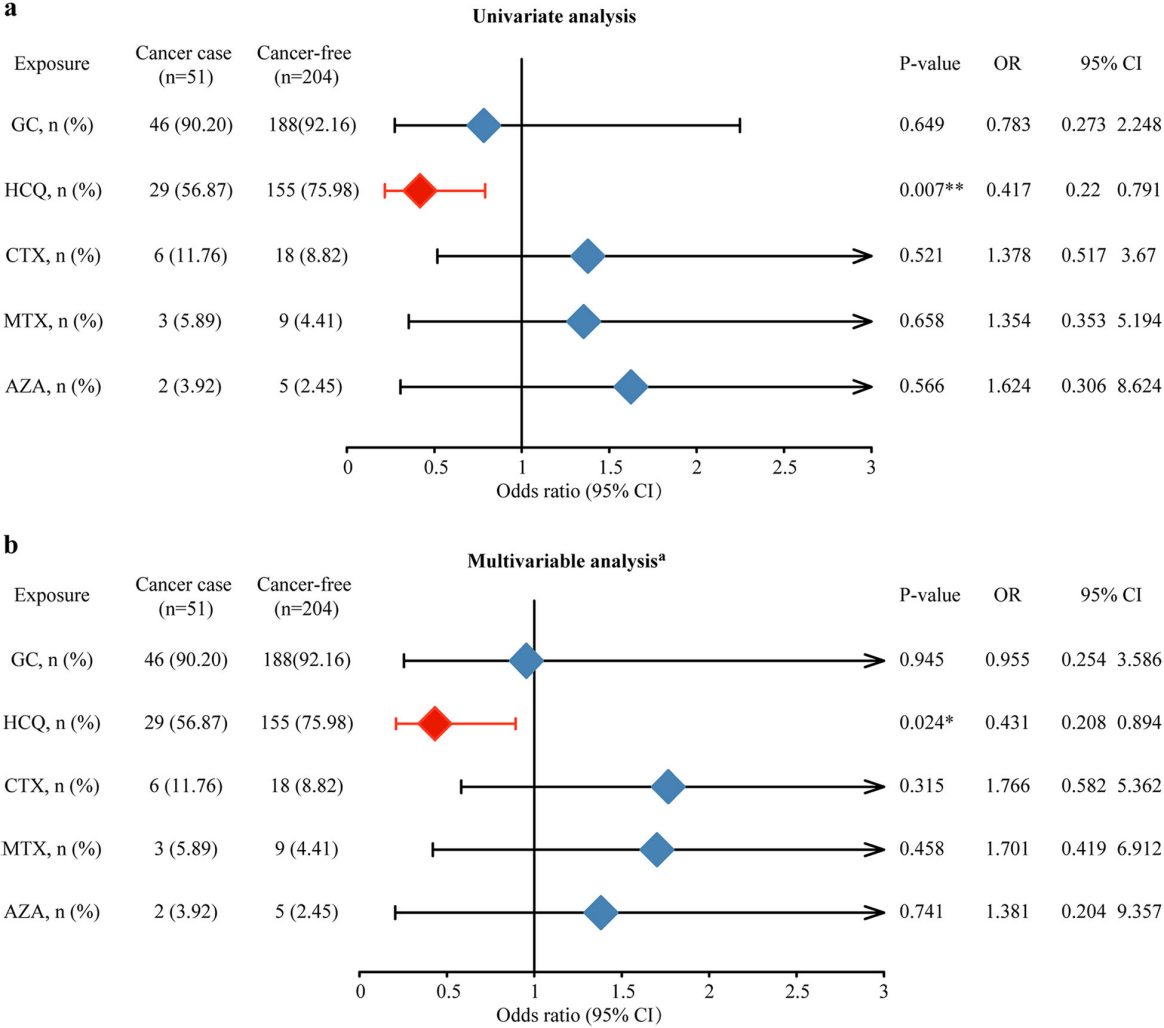
The effect of medication exposure on cancer risk in patients with SLE. **a** Univariate analysis between medication exposure and cancer risk. **b** Multivariate analysis between medication exposure and cancer risk. GC, glucocorticoid; HCQ, hydroxychloroquine; CTX, cyclophosphamide; MTX, methotrexate; AZA, azathioprine; OR, odds ratio. Superscript lowercase letter “a” indicates adjusted for age, gender, age at SLE diagnosis, disease course of SLE, hypertension, diabetes mellitus, and dyslipidemia

The results of association analysis between medication exposure and cancer odds are provided in Fig. 2 and Additional file 2.

Univariate analysis revealed that HCQ was associated with a lower odds of cancer (OR = 0.417, CI 0.220, 0.791), while GC (OR = 0.783, CI 0.273, 2.248), CTX (OR = 1.378, CI 0.517, 3.670), MTX (OR = 0.788, CI 0.219, 2.831), and AZA (OR = 0.653, CI 0.141, 3.014) was not significantly associated with cancer odds. The results were not changed after adjustment for confounding variables.

## Discussion

Numerous studies have demonstrated that patients with SLE had an increased overall cancer risk compared with the general healthy age and sex matched population, especially non-Hodgkin’s lymphoma, thyroid cancer, lung cancer, and vulva cancer. The mechanism remains unclear, it is speculated that various factors including medication exposure, the activated auto-immune system, viral infection, and overlap syndrome as well as traditional lifestyle cancer risk factors may all contribute to the increased cancer risk in SLE [6, 7]. To the best of our knowledge, only a handful of studies have been done to explore the association between cancer and the drugs used in SLE, and the results were inconsistent [18–21]. In this large nested case-control study, we found that the SLE patients with cancer had lower disease activity and that HCQ was negatively associated with cancer risk in SLE patients.

Both SLE and cancer have been associated with immune dysfunction [24]. In SLE patients, the impaired immune system is not able to discriminate between self and non-self-antigens, leading to aberrant production of autoantibodies causing host tissue damage. On the contrary, cancer formation is caused by compromised host’s immune system that cannot recognize cancer antigens. It has been previously reported that the immunogenicity of cancer cell could induce the production of a wide range of auto-antibodies including ANA, anti-dsDNA, anti-Sm, anti-SSA, anti-SSB, anti-Rib, and anti-nRNP [25]. The level of ANA has been reported to be elevated in 31.5% lymphoma patients relative to the control group [12]. While it is well established that anti-dsDNA is highly specific for SLE, it has been found in patients with different malignancies and may serve as a prognostic indicator for cancer. The association between anti-dsDNA and cancer was firstly demonstrated in bronchogenic carcinoma [26]. One study suggested that this antibody may play a role in the pathogenesis of lymphoma and thymoma [27]. It has been hypothesized that the presence of anti-dsDNA autoantibodies in patients with colorectal cancer might indicate better disease outcome [15]. Our current study evaluated the significance of disparities in the levels of ANA, anti-dsDNA, anti-Sm, anti-RO52, anti-RO60, anti-SSB, anti-Nuc, anti-His, anti-Rib, and anti-nRNP antibodies between the cancer group and the cancer-free control group at the time of cancer diagnosis. Our results demonstrated there was no significant difference in the levels of these factors between the cancer group and the control group at the time of cancer diagnosis.

The organ damage and disease activity in SLE patients with or without cancers were investigated, and the results were inconsistent, likely due to the differences in inclusion criteria, race, and scoring systems. For instance, a mean SLICC/ACR damage score of 1.9 and 1.7 has been reported for the cancer group and the control group respectively, suggesting that organ injury was more severe in the cancer group [18]. A different study did not find statistically significant differences in the adjusted mean SLEDAI-2 K between a lymphoma group and a control group [19]. It should be noted that while SLICC/ACR mainly evaluates organ damage [28], the adjusted mean SLEDAI-2 K reflects the mean disease activity after onset [29]. Our results indicate that SLE with different malignancies had lower SLEDAI scores, lower rates of renal involvement, and low level of complement compared with the control group. The SLEDAI mainly reflects the disease activity within 10 days [23]. Taken together, these data indicate that SLE patients with cancers have lower disease activity at the time of cancer diagnosis.

The role of immunosuppressant in cancer development in SLE patients remains controversial. One study showed that immunosuppressant therapy was not associated with overall cancer risk in patients with SLE but might contribute to an increased risk of hematological malignancy [18]. A different research reported that exposure to CTX might contribute to a higher lymphoma risk in SLE patients [19], although this was contradicted by a different report showing that the use of CTX and AZA did not contribute to lymphoma risk [20]. It has been demonstrated that CTX increases cancer risk in SLE patients in a dose-dependent manner [21]. Therefore, more investigations looking at a larger number of participants are needed. In fact, numerous studies have demonstrated that the activated auto-immune system may contribute to the increased cancer risk in patients with SLE, especially non-Hodgkin’s lymphoma. The probable mechanism is the detective immune surveillance system. By virtue of the disease, SLE patients have impaired immune surveillance system due to the activated auto-immune system. In healthy immune system, aberrant cells produced during cell replication are eliminated to prevent them from becoming malignant. In SLE patients, this regulation process may be impaired, making patients more vulnerable to develop cancers. At the same time, the abnormal apoptotic process inherent in SLE may enhance this process [24, 30]. In the scenario of lymphomas occurring in SLE patients, besides the mechanism mentioned above, there is a further aspect that has to be taken into account: these malignancies arise from the immune system itself. The activated lymphocytes in SLE are prone to potentially dangerous genetic events during their maturation, such as recombination or hypermutation in B cells, which eventually promote the development of lymphoma, particularly non-Hodgkin’s lymphoma [31, 32].

HCQ is extensively used in SLE treatment. Besides its well-established effects on the skin and joint symptoms, several studies have indicate that HCQ has important long-term effects on lupus, including reduced long-term accrual damage and decreased long-term mortality [33, 34]. A protective function of antimalarial against cancer in SLE patients has been proposed [35]. Hsu et al. found that HCQ decreased cancer risk in a dose-dependent manner [21]. Our current large-scale study has also elucidated a negative association between HCQ and cancer.

It has been proposed that HCQ might modulate autophagy by impacting lysosomal acidification and blocking the fusion of auto-phagosomes with lysosomes [36]. Chloroquine may trigger the expression of Tp53 which may protect the cells from genotoxic stimuli [37]. In addition, the antimalarial may inhibit unlimited replication of cancer cells via their strong DNA intercalating properties [38]. Chloroquine may promote DNA repair following DNA damage as a result of alkylating therapy [39]. Multiple preclinical and clinical trials have demonstrated a synergistic anticancer effect of HCQ with chemotherapies and targeted therapies [40]. For instance, cytotoxicity of tamoxifen against breast cancer cells has been shown to be enhanced by combination therapy with HCQ [41]. In addition, HCQ is effective against hepatocellular carcinoma and pancreatic ductal adenocarcinoma [42, 43], as well as hematologic cancers like chronic myeloid leukemia, myeloma, and lymphoma [44–46]. Taken together, these reports suggest that HCQ may decrease the cancer risk in SLE patients.

In the SLE cohort included in this study, thyroid cancer, cervical cancer, and lung cancer were the top three cancer types. Studies suggest an increased risk of cervical cancer among SLE patients compared with the general population [47, 48]. It has been reported that immunosuppressant increases the risk of cervical neoplasia in SLE patients and this is attributable to decreased HPV clearance [47, 49]. This suggests that SLE patients under immunosuppressive agents should undergo regular screening for cervical dysplasia.

This retrospective study of a large cohort of SLE patients examined the odds of being diagnosed with cancer in SLE patients. Our results suggest that SLE patients with cancers have lower disease activity at the time of cancer diagnosis. In addition, a negative association between HCQ administration and cancer risk in SLE patients was unveiled, highlighting a novel potential cancer prevention strategy for SLE patients.

## Conclusions

Our analyses indicate that SLE patients with cancers might have a lower disease activity at the time of cancer diagnosis. HCQ was negatively associated with cancer risk in SLE patients. These findings highlight a potential and novel prevention strategy for SLE.

## Supplementary information

**Additional file 1.** Ethical approval certification of this study.

**Additional file 2.** Association between medication exposure and cancer risk.

## Data Availability

The dataset analyzed in this paper is available from the corresponding author on reasonable request, and with appropriate additional ethical approvals, where necessary.

## Abbreviations

SLE: Systemic lupus erythematosus
HCQ: Hydroxychloroquine
ANAs: Antinuclear antibodies
anti-dsDNA: Anti-double-stranded DNA antibody
anti-Sm: Anti-Sm antibody
GC: Systemic glucocorticoid
CTX: Cyclophosphamide
MTX: Methotrexate
AZA: Azathioprine
SLICC/ACR: Systemic Lupus International Collaborating Clinics/American College of Rheumatology
SLEDAI-2 K: SLE Disease Activity Index 2000
ANA: Antinuclear antibody
anti-Ro52: Anti-Ro52 antibody
anti-Ro60: Anti-Ro60 antibody
anti-SSB: Anti-SSB antibody
anti-Nuc: Anti-nucleosome antibody
anti-His: Anti-histone antibody
anti-Rib: Anti-ribosome antibody
anti-nRNP: Anti-nRNP antibody
SLEDAI: SLE Disease Activity Index
WBC: White blood cell
PLT: Platelet
OR: Odds ratio
