# Supplementary material for: The relationship between cancer and medication exposure in patients with systemic lupus erythematosus: a nested case-control study
Source: Arthritis Res Ther. 2020 Jun 26;22:159. doi: 10.1186/s13075-020-02228-6 (PMC7318532; doi:10.1186/s13075-020-02228-6)
Supplement: Supplementary file 1 — Additional file 1. Ethical approval certification of this study. [file 13075_2020_2228_MOESM1_ESM.pdf]

# 郑州大学第一附属医院

## 科研项目伦理审查委员会审查证明

伦理审查编号：2019-KY-199

| | | | |
|---|---|---|---|
| 项目 | 系统性红斑狼疮合作并肿瘤的临床特点及危险因素分析 | | |
| 项目来源 | 1. 国家自然科学基金青年基金<br>2. 国家重点研发计划 | | |
| 项目专业 | 风湿免疫科 | 项目负责人 | 刘升云 |
| 审查方式 | 会议审查 | 审查日期 | 2019 年 07 月 10 日 |
| 审查文件清单 | 审查文件：<br>1. 研究方案（版本号：第一版， 版本日期：2019 年 06 月 01 日）<br>2. 知情同意书豁免声明<br>3. 主要研究者资质 | | |
| 审查意见 | 本项目已通过伦理委员会审查。 | | |

**注：该审查证明仅用于科研人员临床科研目的，不用作产品上市、注册等商业目的。**

郑州大学第一附属医院科研和临床试验伦理委员会

2019 年 07 月 15 日
