# Supplementary material for: The relationship between cancer and medication exposure in patients with systemic lupus erythematosus: a nested case-control study
Source: Arthritis Res Ther. 2020 Jun 26;22:159. doi: 10.1186/s13075-020-02228-6 (PMC7318532; doi:10.1186/s13075-020-02228-6)
Supplement: Supplementary file 2 — Additional file 2. Association between medication exposure and cancer risk. [file 13075_2020_2228_MOESM2_ESM.docx]

**Additional file 2: Association between medication exposure and cancer risk**

| Exposure | Cancer case (n=51) | Cancer-free  (n=204) | Univariate analysis | | Multivariable analysis^a^ | |
| --- | --- | --- | --- | --- | --- | --- |
|  |  |  | OR(95% CI) | *P*-value | OR(95% CI) | *P*-value |
| GC, n (%) | 46 (90.20) | 188(92.16) | 0.783(0.273-2.248) | 0.649 | 0.955(0.254-3.586) | 0.945 |
| HCQ, n (%) | 29 (56.87) | 155 (75.98) | 0.417(0.220-0.791) | 0.007 | 0.431(0.208-0.894) | 0024 |
| CTX, n (%) | 6 (11.76) | 18 (8.82) | 1.378(0.517-3.670) | 0.521 | 1.766(0.582-5.362) | 0.315 |
| MTX, n (%) | 3 (5.89) | 9(7.35) | 1.354(0.353-5.194) | 0.658 | 1.701(0.419-6.912) | 0.458 |
| AZA, n (%) | 2 (3.92) | 5(5.88) | 6.624(0.306-8.624) | 0.566 | 1.381(0.204-9.357) | 0.741 |

GC: glucocorticoid; HCQ: hydroxychloroquine; CTX: cyclophosphamide; MTX: methotrexate; AZA: azathioprine. ^a^adjusted for age, gender, age at SLE diagnosis, disease course of SLE, hypertension, diabetes mellitus and dyslipidemia. OR: odds ratio.
